# Pathogenic variants of the *GNAS* gene introduce an abnormal amino acid sequence in the β6 strand/α5 helix of Gsα, causing pseudohypoparathyroidism type 1A and pseudopseudohypoparathyroidism in two unrelated Japanese families

**DOI:** 10.1016/j.bonr.2022.101637

**Published:** 2022-11-10

**Authors:** Yasuhisa Ohata, Haruna Kakimoto, Yuko Seki, Yasuki Ishihara, Yukako Nakano, Kenichi Yamamoto, Shinji Takeyari, Makoto Fujiwara, Taichi Kitaoka, Satoshi Takakuwa, Takuo Kubota, Keiichi Ozono

**Affiliations:** a Department of Pediatrics, Osaka University Graduate School of Medicine, Suita, Japan; b Department of Pediatrics, Kagoshima University Graduate School of Medical and Dental Sciences, Kagoshima, Japan; c The first Department of Oral and Maxillofacial Surgery, Osaka University Graduate School of Dentistry, Suita, Japan; d Department of Cardiovascular Medicine, Osaka University Graduate School of Medicine, Suita, Japan; e Department of Statistical Genetics, Osaka University Graduate School of Medicine, Suita, Japan; f Department of Pediatrics, Hyogo Prefectural Nishinomiya Hospital, Japan

**Keywords:** AHO, Albright's hereditary osteodystrophy, GPCR, G protein coupling receptors;, HGMD, Human Gene Mutation Database, IRB, Institutional review Board, NMD, Nonsense-mediated decay, TSH, Thyroid-stimulating hormone, Pseudohypoparathyroidism 1A (PHP1A), Pseudopseudohypoparathyroidism (PPHP), *GNAS* gene, Gsα, β6 strand/α5 helix

## Abstract

Pseudohypoparathyroidism 1A (PHP1A) and pseudopseudohypoparathyroidism (PPHP) are caused by loss-of-function variants of *GNAS*, which encodes Gsα. We present two unrelated Japanese families with PHP1A and PPHP harboring unreported pathogenic variants of *GNAS* (c.1141delG, p.Asp381Thrfs*23 and c.1117delC, p.Arg373Alafs*31). These variants introduce abnormal amino acids in the β6 strand/α5 helix of Gsα, which interact with G protein coupling receptor (GPCR). We conclude that these variants alter the association of Gsα with GPCR and cause PHP1A or PPHP.

## Highlights

• Reports of *GNAS* variants causing extra amino acid sequences are limited.
• Two cases with extended Gsα mutants showed clinical characteristics of PHP1A/PPHP.
• No change was found in the affinity between mutant Gsα and GDP using I-TASSER.
• I-TASSER and AlphaFold2 suggested the Gsα mutants caused dysfunction with GPCR.
• Prediction by I-TASSER and AlphaFold2 are useful in determination of pathogenicity.

## 1 Introduction

G protein-coupled receptors (GPCRs), including parathyroid hormone (PTH) and thyroid-stimulating hormone (TSH) receptors, are transmembrane receptors. G protein heterotrimers (Gαβγ) are canonical downstream molecules of GPCRs (Oldham et al., 2006). Gα subunit mediates signal transduction from activated GPCRs, and is regulated by the exchange between guanosine diphosphate (GDP) and guanosine triphosphate in the nucleotide-binding pocket (Jones et al., 2011).

Pseudohypoparathyroidism type 1A (PHP1A) is a rare inheritable disease characterized by Albright's hereditary osteodystrophy (AHO) and hormonal resistance, including PTH, TSH, gonadotropins, calcitonin, growth hormone-releasing hormone, and possibly others (Linglart et al., 2018). PHP1A is caused by loss-of-function variants in the imprinted *GNAS*, which encodes Gsα, in the maternal allele (Spiegel, 1990). In contrast, pathogenic variants of the paternal allele result in pseudopseudohypoparathyroidism (PPHP) characterized by AHO without hormonal resistance (Lebrun et al., 2010).

To predict the functional deficiency of a mutant protein, it is important to estimate the three-dimensional mutant protein structure and compare it with that of the wild-type protein. Zhang et al. released a web server “I-TASSER” which we can predict the protein structure without experimental data (Roy et al., 2010; Yang et al., 2015). Recently, Jumper et al. developed another method based on the deep learning system “AlphaFold2” (Jumper et al., 2021), which has shown outstanding performance in the prediction of protein structures.

Several types of pathogenic variants of *GNAS* were found in PHP1A/PPHP, and most cases (66 %) presented with frameshift, nonsense, or splicing variants, which results in truncated mutants or nonsense-mediated decay (NMD) (Rickard and Wilson, 2003). These pathogenic variants are expected to inactivate or induce haploinsufficiency in Gsα function, causing PHP1A or PPHP. Variants that may lead to an extra abnormal amino acid sequence as a pathogenic variant of PHP1A/PPHP are rare, and there are few reports of these cases. In this case report, we describe novel variants in exon 13 of *GNAS* that result in the addition of abnormal amino acid sequences in the β6 strand/α5 helix, predicted using I-TASSER and AlphaFold2, and cause both PHP1A and PPHP in two unrelated Japanese families.

## 2 Case

**Fig. 1:**
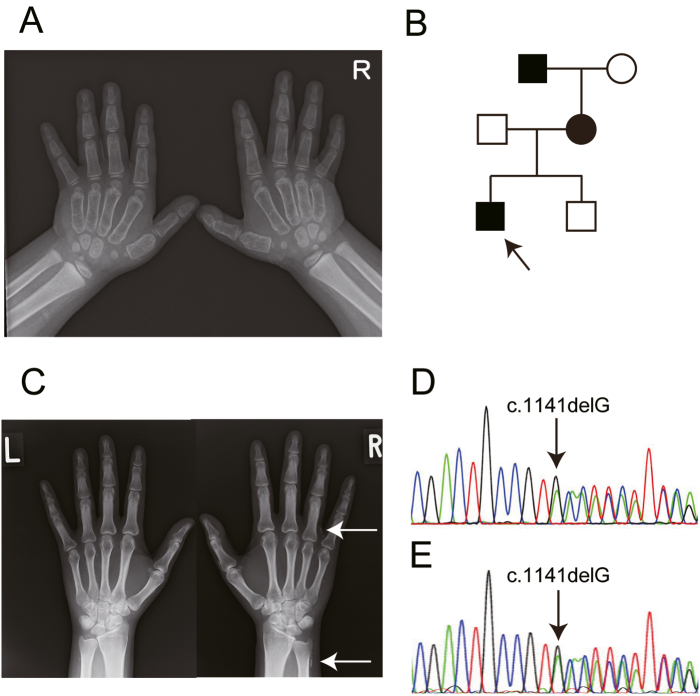
Case 1 family. A. Radiography of the hands of the proband with brachydactyly of metacarpals. B. Pedigree of the family. The black square and circle denote patients and the arrow indicates the proband. C. Radiography of the hands of the mother with brachydactyly of metacarpals IV. The white arrows show the subcutaneous heterotopic ossification. D, E. Sequencing revealed heterozygous c.1141delG variant of *GNAS* both in the proband (D) and the mother (E).

Case 1 involved a 5-year-old boy with subcutaneous heterotopic ossification diagnosed *via* biopsy, who was referred to us because of hypocalcemia and hyperphosphatemia. His height and body weight were 110.8 cm (+0.81 standard deviation [SD]) and 24.2 kg (+28.1 %), respectively. He had AHO symptoms including round and flat face, brachydactyly (Fig. 1), knuckle sign, and mild mental retardation.

We initiated treatment with active vitamin D and thyroid hormone for the proband because laboratory tests showed hypocalcemia, hyperphosphatemia, and hypothyroidism (Table 1).

**Table 1:** Laboratory data of the proband and the mother in case 1.

	Proband	Mother
Calcium (mg/dL)	6.9	9.3
Phosphate (mg/dL)	7.8	3.8
Albumin (g/dL)	4.1	4.7
Intact-PTH (pg/mL)	524.9	45.6
25OHD (ng/mL)	17	22
1,25(OH)_2_D (pg/ml)	85	NA
Creatinine (mg/dL)	0.29	0.65
TSH (μIU/mL)	14.3	2.79
Free T4 (ng/dL)	1.2	1.3
Free T3 (pg/mL)	3.0	2.5

PTH. parathyroid hormone; 25OHD. 25-hydroxyvitamin D; 1,25(OH)_2_D. 1,25-dihydroxyvitamin D; TSH. thyroid-stimulating hormone; T4. thyroxine; T3. triiodothyronine; NA. not available.

The patient's mother and maternal grandfather also had subcutaneous heterotopic ossification (Fig. 1). The mother's height and body weight were 163 cm and 57.4 kg, respectively and she had no AHO symptoms other than brachydactyly in metacarpal IV in the right hand (Fig. 1) or abnormal laboratory data (Table 1). Genetic analysis revealed a heterozygous c.1141delG (p.Asp381Thrfs*23) variant of *GNAS* in the proband and his mother (Fig. 1).

Case 2 involved a 1-year-old boy with subcutaneous heterotopic ossification diagnosed *via* biopsy, who was referred to us. The patient did not have any AHO symptoms, except for ossification at the first visit. His height and body weight were 69.2 cm (−0.53 SD) and 9.3 kg (+0.70 SD), respectively. Laboratory data revealed hypothyroidism (Table 2); therefore, we initiated thyroid hormone treatment.

**Table 2:** Laboratory data of the proband in case 2.

	9 months old	2 years old	3 years old	4 years and 5 months old	4 years and 8 months old
Calcium (mg/dL)	9.9	9.6	9.4	9.0	8.1
Phosphate (mg/dL)	6.6	6.2	5.8	7.5	6.9
Albumin (g/dL)	4.0	4.1	NA	NA	4.4
Intact-PTH (pg/mL)	44	296	299	550	NA
25OHD (ng/mL)	25.8	NA	NA	NA	12.1
Creatinine (mg/dL)	0.29	NA	0.30	0.35	0.31
TSH (μIU/mL)	7.32	10.4	5.46	5.17	5.31
Free T4 (ng/dL)	0.87	0.78	0.91	1.00	0.90
Free T3 (pg/mL)	3.0	NA	2.9	NA	NA

PTH. parathyroid hormone; 25OHD. 25-hydroxyvitamin D; 1,25(OH)_2_D. 1,25-dihydroxyvitamin D; TSH. thyroid-stimulating hormone; T4. thyroxine; T3. triiodothyronine; NA. not available.

**Fig. 2:**
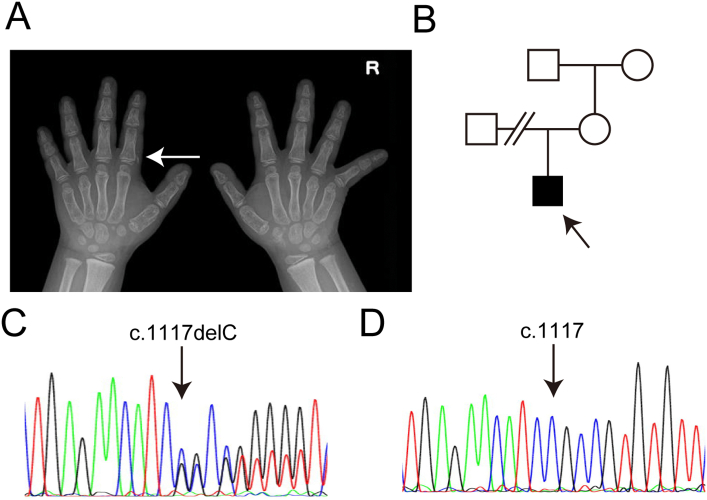
Case 2. A. Radiography of the right hand of the proband with brachydactyly of metacarpals. White arrows show the subcutaneous heterotopic ossification. B. Pedigree of the family. The arrow indicates the proband. C, D. Sequencing revealed a heterozygous c.1117delC variant of *GNAS* in the proband (C), while the variant was not detected in the mother (D).

His mother and other family members showed no PHP1A or PPHP symptoms (Fig. 2). Since PTH resistance and hypocalcemia appeared during the clinical course (Table 2), we initiated additional active vitamin D treatment when the proband was from 4 years and 8 months old. Genetic testing revealed a heterozygous c.1117delC (p.Arg373Alafs*31) variant of *GNAS*, although the mother did not harbor this variant (Fig. 2).

**Fig. 3:**
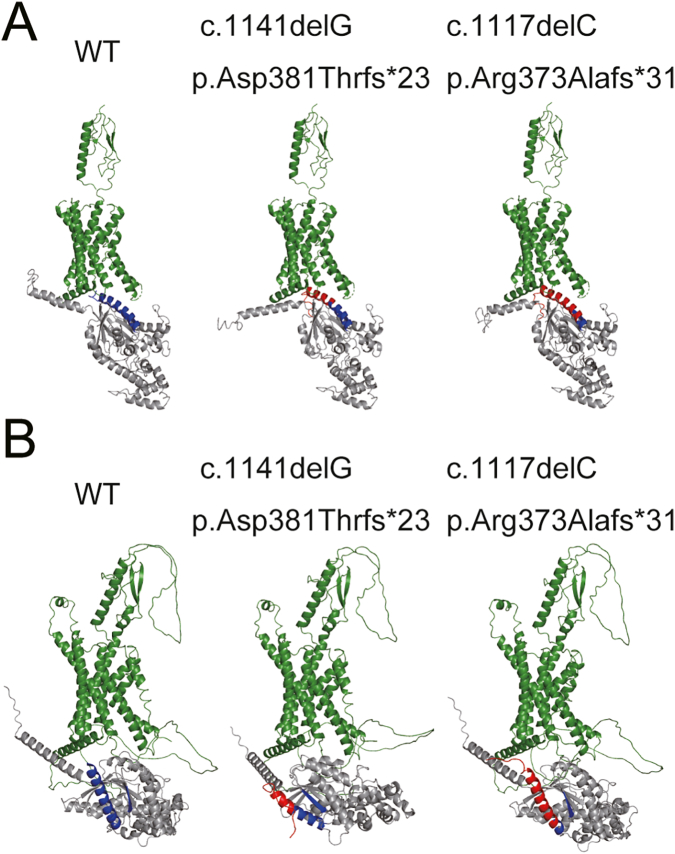
Prediction of the protein structure of Gsα and PTH/PTHrP receptor complex using ribbon diagrams. Gsα is shown in gray, receptor in green. The β6 strand/α5 helix of Gsα is shown in blue and the abnormal amino acid sequence caused by the pathogenic variants are shown in red. A. Prediction using I-TASSER. B. Prediction using AlphaFold2.

These pathogenic variants we detected have not been reported in the relevant databases (Leiden Open Variation Database [https://databases.lovd.nl/shared/genes] and The Human Gene Mutation Database [HGMD: http://www.hgmd.cf.ac.uk/ac/index.php]). To predict the changes in the three-dimensional structure of mutant Gsα, we used I-TASSER (https://zhanglab.ccmb.med.umich.edu/I-TASSER/) (Roy et al., 2010; Yang et al., 2015; Zhang, 2008) and AlphaFold2 (Jumper et al., 2021). These prediction analyses revealed that the frameshift variants introduced an additional 22 or 30 amino acids with abnormal sequences to the β6 strand/α5 helix of Gsα, which interact with the GPCR (Fig. 3).

The estimated affinity score calculated using I-TASSER between the Gsα with and without variants and GDP, which is a substrate for Gsα, showed no significant difference among these groups; the confidence scores of the prediction were 0.87, 0.83, and 0.94 for wild type protein, c.1141delG (p.Asp381Thrfs*23), and c.1117delC (p.Arg373Alafs*31) variants, respectively.

## 3 Discussion

Recently, 69 small deletion variants were reported in HGMD (assessed in April 2022) as the cause of PHP1A (n = 47), AHO (n = 16), progressive osseous heteroplasia (n = 4), multiple congenital anomalies (n = 1), and nephrogenic syndrome of inappropriate antidiuresis (n = 1). Most variants caused frameshift variants, which may lead to premature stop codons and result in truncating mutants or NMD. Elli et al. investigated a large cohort with *GNAS* deficiency (n = 407) and reported that major frameshift variants were distributed in exons 1, 5, and 7 (Elli et al., 2016). The pathogenic variants of p.Asp381Thrfs*23 and p.Arg373Alafs*31, reported here, are located in exon 13, resulting in additional abnormal amino acid sequences in the 3′ region and producing extended mutant proteins. From the HGMD database, we reviewed previous literatures describing pathogenic variants of *GNAS* that lead to a frameshift and produce extended mutants, to which abnormal amino acid sequences are added. We found only two small deletion variants (c.1148_1151delTTCA, p.Ile383Serfs*20; c.1164delT, p.Arg389Valfs*15) (Snanoudj et al., 2020; Thiele et al., 2015), one small insertion variant (c.1097_1108dup12, p.Ala366_Thr369dup) (Aldred et al., 2000), and one gross deletion variant (c.1039-92_1051del105, p.Arg347Profs*53) (Garin et al., 2015), while no indel variant was detected which would result in an additional amino acid sequence. Although the extended mutants are rare, these cases presented here provide evidence that these Gsα mutants result in loss of function and lead to PHP1A/PPHP. Thiele et al. analyzed genotype-phenotype correlations including extended mutants, but no significant correlation was reported because the sample size was too small (Thiele et al., 2015). It is important to accumulate data on such pathogenic variants to clarify the genotype-phenotype correlation.

The American College of Medical Genetics and Genomics has published revised guidelines for the interpretation of sequence variants (Richards et al., 2015). Based on this algorithm, c.1141delG and c.1117delC are designated as “likely pathogenic” because the former variant has PM1 (located in a mutational hot spot and/or a critical and well-established functional domain without benign variation) + PM2 (absent from controls [or at extremely low frequency if recessive] in the Exome Sequencing Project, 1000 Genomes or ExAC) + PP1 (co-segregation with disease in multiple affected family members in a gene definitively known to cause the disease) + PP4 (patient's phenotype or family history is highly specific for a disease with a single genetic etiology), and the latter has PS2 (*de novo* [both maternity and paternity confirmed] in a patient with the disease and no family history) + PM1 + PM2. If *in vitro* functional studies reveal a damaging effect, PS3 (Well-established *in vitro* or *in vivo* functional studies supportive of a damaging effect on the gene or gene product) can be added to both variants and their pathogenicity prediction will be changed to “pathogenic.” Since protein structure prediction analysis contributes to *in vitro* mutant functional assays, it is important to determine pathogenicity.

In case 2, PTH resistance gradually became obvious during early childhood. Usardi et al. reported that patients with *GNAS*-inactivating variants of the maternal allele were diagnosed at a mean age of 3.9. They also reported that their patients showed a normal increase in urinary cyclic adenosine monophosphate (cAMP) using the Ellsworth-Howard test at the age of 7 months, while the response was diminished by the age of 3.9 years old (Usardi et al., 2017). Although it is a limitation that we could not obtain a genetic analysis for the father of case 2 proband due to separation, and could not confirm the origin of the pathogenic variant, we believe that it was *de novo* and localized on the maternal allele since he showed typical clinical features of PHP1A as a grown up.

In both cases, subcutaneous heterotopic ossification was the first symptom. Pignolo et al. reported that heterotopic ossification results from a decrease in cAMP signaling and subsequent dysregulation of lineage switching between osteoblast and adipocyte fate, which accelerates osteogenic differentiation in adipose-derived mesenchymal progenitor cells (Pignolo et al., 2011). It can be expected that these mutants, which we have reported here, decrease the cAMP signaling through a certain mechanism. Since no change was revealed in the affinity prediction analysis between mutants and the substrate, we concluded that the mutants led to the loss of function without affecting on substrate affinity. Structural prediction using I-TASSER and AlphaFold2 showed that the mutant caused the destruction of helix structure at the end of the α5 helix. The α5 helix was found to be far from the GPCR in 3D structures, especially in the p.Asp381Thrfs*23 variant, as revealed by AlphaFold2 analysis (Fig. 3). Although the prediction of missense variants using AlphaFold2 remains controversial (Buel and Walters, 2022), analysis of our frameshift variants revealed the abnormal sequence facing to the GPCR as I-TASSER did. Chung et al. reported that the agonist-activated GPCR engages the C terminus of the α5 helix of Gsα which undergoes a rigid-body translation upward into the receptor core and reorganizes the β6 strand/α5 helix, a region that participates in purine ring binding to transduce the signal (Chung et al., 2011). These prediction analyses suggest that the abnormal sequence interfered with the interaction between the GPCR and mutant Gsα, resulting in dysfunction. Although further studies are warranted to clarify the precise pathogenesis mechanism, these predictive analyses can help reveal the pathophysiology.

There is a limitation of this study. We have shown no evidence that the Gsα mutants, reported here, are expressed in the cells. Makita et al. reported the mutant Gsα harboring AVDT amino acid repeats caused PHP1A because of the instability of the mutant Gsα (Makita et al., 2007). From these data, it is possible that the pathogenesis of the variants of *GNAS*, we described here, is also originated from the instability of mutant Gsα. We need to prove the expression of mutant Gsα with extra abnormal amino acid to verify the predicted pathophysiology based on the predictive analyses.

In conclusion, this case report supports the evidence that an extra abnormal amino acid sequence at the β6 strand/α5 helix of Gsα can cause PHP1A/PPHP. Structural prediction using I-TASSAR and AlphaFold2 helped predict the pathogenic mechanism, although further studies are required to elucidate the precise mechanisms.

## CRediT authorship contribution statement

**Yasuhisa Ohata:** Conceptualization, Data curation, Formal analysis, Investigation, Validation, Visualization, Writing – original draft. **Haruna Kakimoto:** Data curation. **Yuko Seki:** Data curation. **Yasuki Ishihara:** Data curation, Visualization. **Yukako Nakano:** Data curation. **Kenichi Yamamoto:** Investigation, Data curation. **Shinji Takeyari:** Data curation, Investigation. **Makoto Fujiwara:** Data curation, Writing – review & editing. **Taichi Kitaoka:** Data curation, Writing – review & editing. **Satoshi Takakuwa:** Data curation. **Takuo Kubota:** Writing – review & editing. **Keiichi Ozono:** Funding acquisition, Supervision, Writing – review & editing.

## Declaration of competing interest

Authors do not have any conflicts of interest to report.

## Data Availability

Data will be made available on request.
